# An effective nano drug delivery and combination therapy for the treatment of *Tuberculosis*

**DOI:** 10.1038/s41598-022-13682-4

**Published:** 2022-06-10

**Authors:** Mojgan Sheikhpour, Vincent Delorme, Alibakhsh Kasaeian, Vahid Amiri, Morteza Masoumi, Mohammad Sadeghinia, Nayereh Ebrahimzadeh, Mobina Maleki, Shahin Pourazar

**Affiliations:** 1 Department of Mycobacteriology and Pulmonary Research, Pasteur Institute of Iran, Tehran, Iran; 2 Microbiology Research Center, Pasteur Institute of Iran, Tehran, Iran; 3 Tuberculosis Research Laboratory, Institute Pasteur Korea, Seongnam, Gyeonggi Republic of Korea; 4 Faculty of New Science and Technology, University of Tehran, Tehran, Iran; 5 School of Chemistry, University College of Science, University of Tehran, Tehran, Iran

**Keywords:** Biotechnology, Microbiology, Drug delivery, Pharmaceutics

## Abstract

Drug resistance in tuberculosis is exacerbating the threat this disease is posing to human beings. Antibiotics that were once effective against the causative agent, *Mycobacterium tuberculosis* (Mtb), are now no longer usable against multi- and extensively drug-resistant strains of this pathogen. To address this issue, new drug combinations and novel methods for targeted drug delivery could be of considerable value. In addition, studies have shown that the use of the antidepressant drug fluoxetine, a serotonin reuptake inhibitor, can be useful in the treatment of infectious diseases, including bacterial infections. In this study, an isoniazid and fluoxetine-conjugated multi-walled carbon nanotube nanofluid were designed to increase drug delivery efficiency alongside eliminating drug resistance in vitro. The prepared nanofluid was tested against Mtb. Expression levels of *inhA* and *katG* mRNAs were detected by Real-time PCR. ELISA was applied to measure levels of cytokine secretion (TNF-α, and IL-6) from infected macrophages treated with the nano delivery system. The results showed that these nano-drug delivery systems are effective for fluoxetine at far lower doses than for free drugs. Fluoxetine also has an additive effect on the effect of isoniazid, and their concomitant use in the delivery system can have significant effects in treating infection of all clinical strains of Mtb. In addition, it was found that the expression of isoniazid resistance genes, including *inhA, katG*, and the secretion of cytokines TNFα and IL6 under the influence of this drug delivery system is well regulated. It was shown that the drug conjugation can improve the antibacterial activity of them in all strains and these two drugs have an additive effect on each other both in free and conjugated forms. This nano-drug delivery method combined with host targeted molecules could be a game-changer in the development of a new generation of antibiotics that have high therapeutic efficiencies, low side effects, and the potential to overcome the problem of drug resistance.

## Introduction

According to the WHO reports, tuberculosis (TB) is among the top ten annual major causes of fatality and morbidity worldwide^1^. The disease is mainly caused by *Mycobacterium tuberculosis* (Mtb), a human pathogen that has been evolving to acquire resistance to drugs, leading to the emergence of multidrug-resistant (MDR) and extensively drug-resistant (XDR) phenotypes^2,3^. To prevent the further spread of such strains, novel approaches to diagnosis and treatment are intensely needed.

Isonicotinic acid hydrazide or isoniazid (INH) has a strong antimycobacterial activity and is being used in the clinic as the main first-line agent for decades. The treatment however suffers from its length and presence of side effects. The emergence of bacterial resistance is also a detrimental issue that should be addressed to maintain the usability of this life-saving drug^4–7^. Two genes that are highly involved in INH resistance are *katG* and *inhA*, with the former coding for KatG, responsible for INH bio-activation^8^, and the latter coding the target of the INH metabolite^9^. Therefore, resistance to INH in Mtb is associated with mutations within these two genes^10,11^. Rapid sequencing of INH-resistant strains showed significant differences in mutation rates from country to country. Differences in *katG* and *inhA* gene mutation rates between Beijing strains and non-Beijing strains could explain such discrepancies. For example, reverse hybridization studies successfully detected over 80% of INH-resistant strains among Korean isolates. Similar studies indicated that mutations in these two genes (*katG* and *inhA*) were found in 89.3% of the MDR and XDR isolates in Shanghai (China), 64% in Africa, and about 70–90% in Iran^12–16^.

Assessment of different antidepressant drugs including fluoxetine (FLX) revealed the antibacterial feature of CNS drugs, when used alone or in combination with different antibiotics, mainly through inhibition of efflux pumps^17^. Likewise, the reported synergic effects of FLX with gentamycin and erythromycin in treating MDR strains of *Pseudomonas aeruginosa* and *Escherichia coli* illustrate not only its antimicrobial activity but also its antibiotic modulatory role^18^. In a recent review article, the collective impact of FLX with other antiviral drugs has been proposed as a promising treatment and preventative approach for coronavirus disease^19^.

FLX is an approved serotonin reuptake inhibitor upregulating serotonin levels through agonistic activity toward 5-HT2B^20^, as well as 5-HT2C receptors^21^, both belonging to the G- protein-coupled receptor family. When used in macrophages infected with Mtb, FLX was found to inhibit mycobacterial growth by enhancing TNF-α secretion and inducing autophagy in infected macrophages^22^.

In terms of targeted drug delivery, conjugation of drugs with carbon nanotubes (CNTs) has drawn much attention with regards to their potential use in the diagnosis and therapy of different diseases^23–25^. To circumvent multidrug resistance in bacteria and reduce the dosage of drugs, nano-drug delivery via functionalized CNTs can be utilized. Studies have shown that both single-walled CNT (SWCNT) and multi-walled CNT (MWCNT) were able to hinder multidrug resistance, exerting their antibacterial effect through cell wall destruction, induction of oxidative stress, and fracturing of the bacterial DNA or macromolecules^26^. This CNT system can also be formulated in the form of nanofluids or nanoparticle suspension as they have high dispersion stability and bioavailability, and, therefore, are a viable method for drug delivery. Besides, the recommended therapeutic drugs for the treatment of TB have various neurotoxicity and hepatotoxicity side effects. So, Nano drug delivery systems of these drugs have extensive potential to solve these problems via improvement of the bioavailability of drugs and reducing the dosage and frequency of administration^27,28^.

In our previous study, we developed a nano-drug delivery system for INH that displayed a higher antimicrobial effect in vitro against the reference laboratory strain of Mtb (H37Rv) compared to the pure drug^29^. The primary goal of this study was to investigate the possible antibacterial effect of the FLX and INH combination when used as a synthesized nano-drug delivery system, as compared to the pure drugs used in combination or alone on three different strains of Mtb: the reference laboratory strain H37Rv and two clinical isolates classified as MDR or XDR. In addition, our study aimed at exploiting MWCNTs, which have lower toxicity compared to SWCNTs^30^. Further, the gene expression profile of *inhA* and *katG* in all treated groups were captured to understand the influence of these drug formulations on bacterial resistance. Finally, variations in the level of TNF-α and IL-6, both pivotal markers of TB-induced inflammation, were analyzed in response to the treatment.

## Materials and methods

### Preparation of INH, FLX, and MWCNTs

4-pyridine carboxylic acid hydrazide (INH) was purchased from Darou Pakhsh Pharmaceutical Co. (Tehran, Iran). Carboxylated (-COOH) MWCNTs were purchased from Neutrino Co. (Tehran, Iran) with specifications as presented in Table 1. Fluoxetine (FLX) was purchased from Abidi Pharmaceutical Co. (Tehran, Iran).

**Table 1:** Specifications of carboxyl multi-walled carbon nanotubes (MWCNT-COOH).

Purity	> 95%
-COOH content	3.86 wt%
The rate of surface carbon atom	8–10 mol%
Outer diameter	< 8 nm
Inner diameter	2–5 nm
Length	10–30 µm
Special surface area	> 500 m^2^/g
Color	Black
Tap density	0.27 g/cm^3^
True density	~ 2.1 g/cm^3^
Electric conductivity	> 100 s/cm
Manufacturing method	CVD

### Chlorination of functionalized MWCNTs

A suspension of 200 mg MWCNT in 20 ml thionyl chloride (Merck, Germany) was refluxed for 14 h at 60 °C in a chemical hood. Then the chlorinated (COCl) MWCNT was dried at 60 °C under the hood.

### Drug conjugation

For loading the drug on CNT, a suspension of 100 mg chlorinated MWCNT and 300 mg drug (ratio 1:3 w/w) in 20 ml dimethylformamide (DMF) (Merck, Germany) was stirred for 20 min before sonication at 200 W in an ultrasonic device. The obtained fluid was incubated at room temperature for 6 h, then refluxed at 70° C for 16 h. This was followed by several stages of centrifugation (at 8000 rpm, 15 min each time) and washing with tetrahydrofuran, methanol, and ethanol (96%). Finally, the precipitate was dried at room temperature.

### Nano-drug characterization

To ensure the binding of the drug on the CNT, elemental analysis (C-H-N-S) and Fourier transform infrared (FT-IR) analysis tests were carried out. Conjugated nanotubes and nanotubes alone were also observed and analyzed under scanning electron microscopic observation (SEM).

### Nanofluid preparation

To a suspension of 200 mg nano-drug powder in 6 mL of ethanol (96%) was added 60 mg of Arabic gum dissolved in 100 mL of deionized water and the resulting mixture was stirred for 20 min. The mixture was then placed on ice and sonicated for 20 min at 200 W.

### Stocks preparation and microbial testing

Mtb strains including the reference laboratory strain H37Rv, together with an MDR, and XDR clinical isolates were obtained from the microbial bank of the Mycobacterium and Pulmonary Research Department, Pasteur Institute, Iran. For each strain, microbial suspension of 0.5 McFarland turbidity (equal to 1.5 × 10^8^ bacteria) was prepared and bacterial strains were cultured in a Lowenstein-Jensen culture medium.

Initial stocks of INH, FLX, MWCNT-INH and MWCNT-FLX were prepared with the first concentration of 1 mg/mL, 1 mg/mL, 2 mg/mL, and 2 mg/mL respectively. Then different dilutions of INH and FLX were prepared with the first concentration of 143, and 40 µg/mL respectively. MWCNT-INH and MWCNT-FLX were prepared, starting from an initial concentration of 100 and 111 µg/mL respectively. The summary of all dilutions and the corresponding concentrations is presented in Table 2.

**Table 2:** Dilutions and concentrations of pure and conjugated drugs used in this study.

Dilution	Concentration (µg/mL)			
	INH	FLX	MWCNT-INH	MWCNT-FLX
40	–	1600	–	–
20	–	800	–	–
10	–	400	–	–
5	–	200	–	–
2	–	80	–	222
1	143	40	100	111
1/2	71.5	20	50	55.5
1/4	35.75	10	25	27.75
1/8	17.87	5	12.5	13.87
1/16	8.93	2.5	6.25	6.93
1/32	4.47	1.25	3.125	3.47
1/64	2.23	–	1.56	1.73
1/128	1.12	–	0.78	0.87
1/256	0.56	–	0.39	0.43
1/512	0.28	–	0.19	0.22
1/1024	0.14	–	–	–
1/2048	0.07	–	–	–

Evaluation of the drug effects and their dose-dependencies were conducted on the bacterial strains using both the pure and conjugated forms of the drugs. At first, for the determination of growth, a microplate-based assay that uses Alamar blue reagent was evaluated for all studied concentrations. Then each obtained results of MICs against Mtb H37Rv, MDR, and XDR strains were validated by tube assay test. A checkerboard assay was performed for the combination of MWCNT-INH and MWCNT-FLX. Observations were made based on the growth of bacteria after two to three weeks of incubation.

### Evaluation of synergy

The fractional inhibitory concentration (FIC) index was calculated to characterize quantitatively the activity of the antimicrobial combinations. The FIC represents the sum of the FICs of each drug tested. The calculation was done based on the following equation: A/ MIC_A_+ B/ MIC_B_ = FIC_A_ + FIC_B_ = FIC Index, where A and B are the MIC of each antibiotic in combination (in a single well), and MIC_A_ and MIC_B_ are the MIC of each drug individually^31,32^. The FIC index value is then used to categorize the interaction of the INH and FLX, and also MWCNT-INH and MWCNT-FLX as follow:

FIC value < 0.5 = synergy, > 4 = antagonism, and 0.5–4 = Additive or indifference.

### Cell culture

Experiments were carried out with A549 and THP-1 human cell lines, which were provided by the National Cell Bank of Iran (NCBI) (Pasteur Institute, Iran). A549 and THP-1 cells were cultured at 37° C in DMEM and RPMI, respectively, supplemented with 10% of fetal bovine serum (FBS). The cells were seeded in 6 well plates and THP-1 cells differentiated in macrophages with PMA at a concentration of 50 ng/µL for one day.

### In vitro Mtb infection and cellular treatments

The supernatant was removed from each well and the cells were washed with PBS, followed by the addition of a fresh culture medium. Cells were infected for 4 h at 37 °C, at a multiplicity of infection (MOI) of 10, with a suspension of the MDR, XDR, or the H37Rv bacterial strain, adjusted to a turbidity of 0.5 McFarland with RPMI medium.

Based on the results obtained from the tube-tests studies, further evaluations such as gene expression and cytokine quantification were performed. The cells were treated with INH, FLX, MWCNT-INH, MWCNT-FLX, and a combination of drugs and nano-drugs. In each 6 well plates, the different doses (1/512, 1/1024, 1/2048) of the INH, (1/32, 1/64, 1/128) of MWCNT-INH, (60, 40) of FLX, and (2, 4, 8) of MWCNT-FLX were repeated twice. At the end of the incubation period, the pellets and supernatants of treated cells were collected for molecular and cytokines studies.

### Analysis of gene expression

RNA extraction was performed for cells treated with doses less than the obtained MIC value of each group. Total RNAs were extracted from tubes in which bacteria had less growth than the control using the Gene all kit (Cat. No. 301-001) according to the manufacturer's protocol. Total RNA was transcribed into single-stranded cDNA using the Easy™ cDNA Synthesis Kit (Pars Tous, Iran). Finally, Real-Time PCR was carried out using specific primer pairs as shown in Table 3.

**Table 3:** Primer sequences used for RT-PCR.

Target gene	Forward primer 5′-3′	Reverse primer 5′-3′	Product (bp)
*katG*	AGAAGAAGTACGGCAAGAAG	TACCGCTGTAACGCTCAT	183
*inhA*	CATCTCGGCGTATTCGTA	CGTCATCCAGTTGTAGGC	124
*16s rRNA*	AAGTCGGAGTCGCTAGTA	TACGGCTACCTTGTTACG	183

### Cytokine quantification and statistical analysis

ELISA was performed on supernatant collected 24 h after treatment to quantify the secretion of IL6 and TNF-α cytokines using specific kits (IBL International, Germany). The data were organized in Excel and analyzed by ANOVA using Prism software (GraphPad). Results were considered significant when P-value was lower than 0.05.

## Results

### Characterization of the nanosystems

#### Elemental analysis

The results of the elemental analysis conducted to ensure the binding of drugs to the carbon nanotubes can be seen in Table 4. Naked carbon nanotubes do not contain any nitrogen, but detectable amounts were seen in drug-functionalized nanotubes, indicating effective binding of the drugs to the carbon nanotubes. Considering the mass content of azote (N) in 100 g of MWCNT, a loading of 0.6 mmol INH/g can be inferred for MWCNT-INH, and 1.4 mmol FLX/g for MWCNT-FLX. Because of minor CNT mass reduction due to centrifugation and washing, the carbon balance could not be used to estimate the loading.

**Table 4:** CHNS elemental analysis results of isoniazid and fluoxetine nano systems.

Sample name	%C	%H	%N	%S
MWCNT	96.40	0.04	0.00	0.00
INH	60.00	6.02	34.70	0.00
MWCNT-INH	90.71	0.62	2.66	0.00
FLX	61.00	5.70	4.26	0.00
MWCNT-FLX	71.28	0.51	1.29	0.00

### Comparison of infrared spectroscopy of non-functionalized nanotubes with drug-activated nanotubes

**Figure 1:**
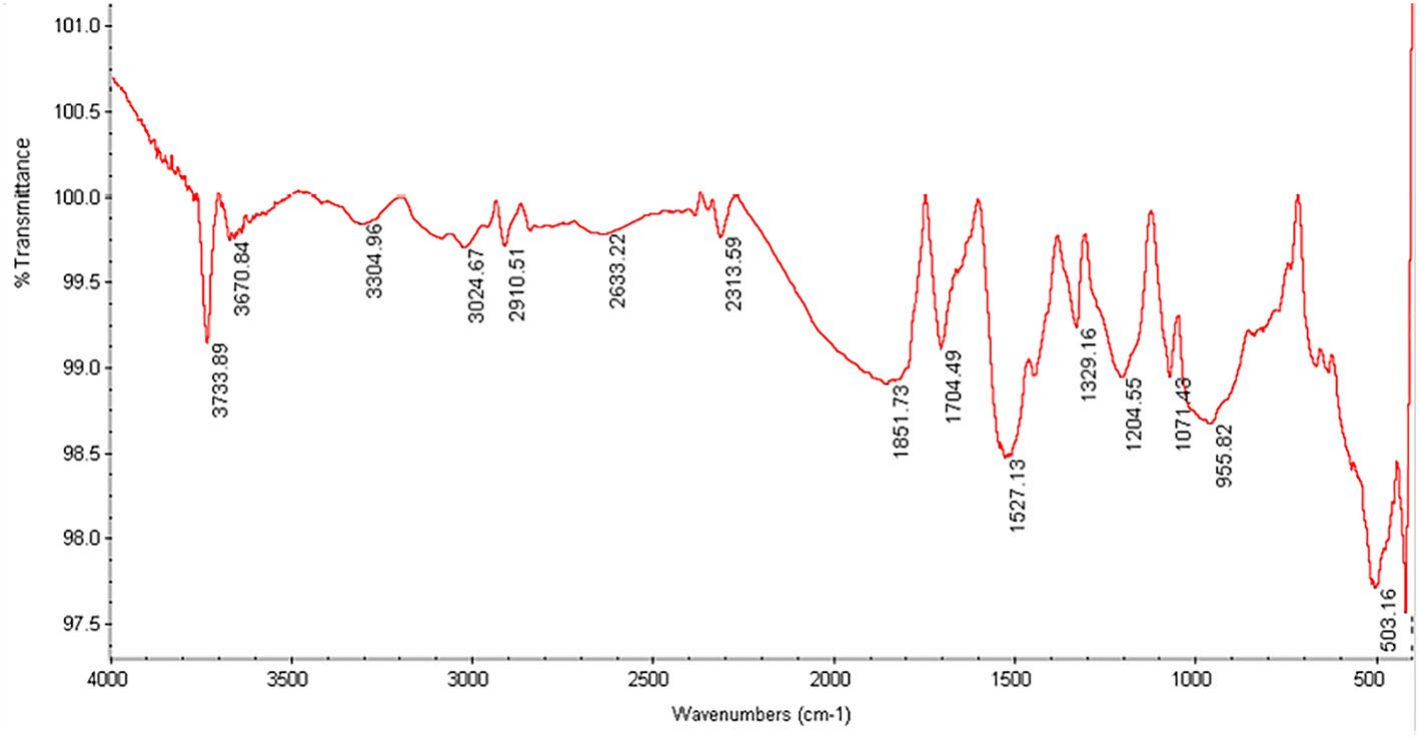
The infrared spectrum of carboxylated nanotubes.

**Figure 2:**
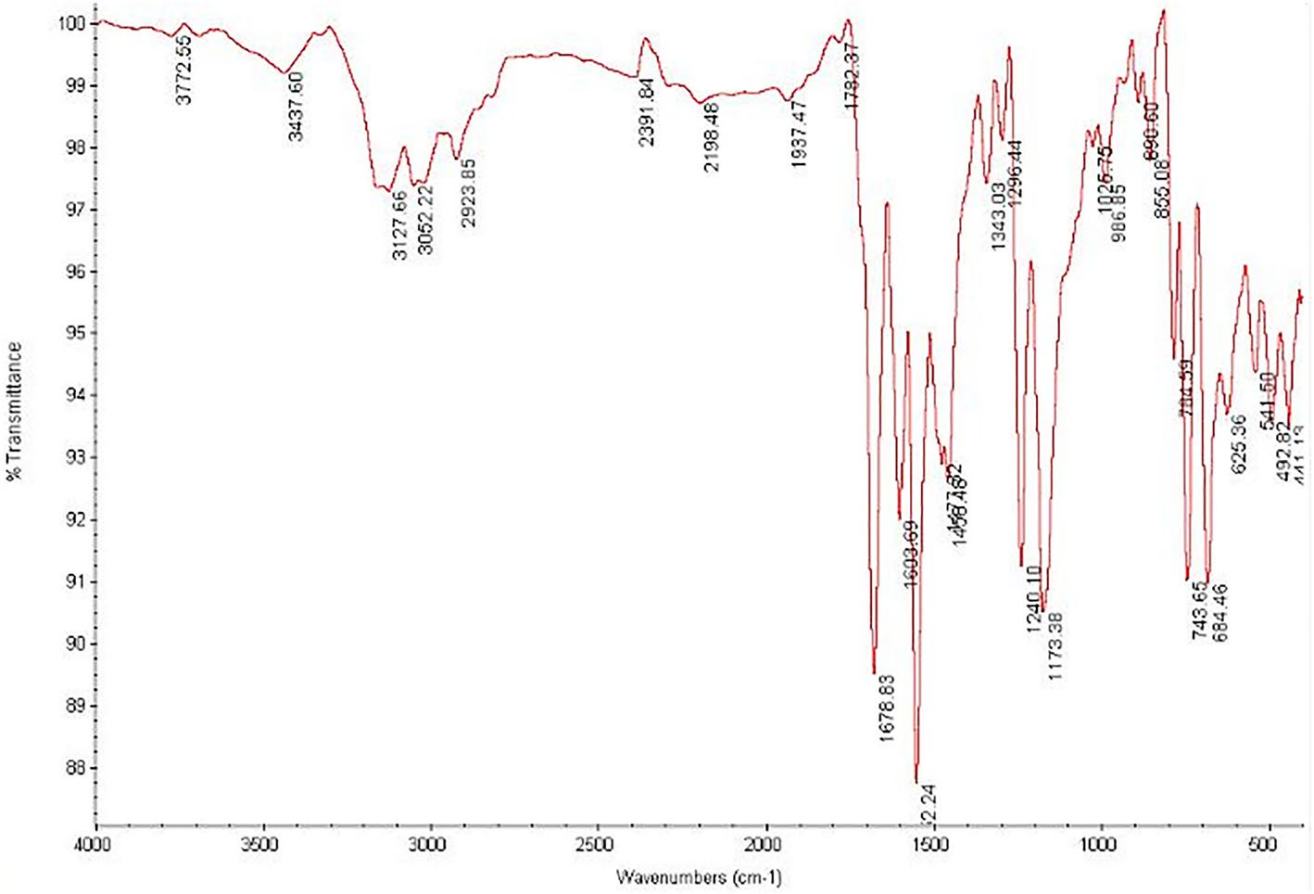
The infrared spectrum of nanotubes functionalized with INH.

**Figure 3:**
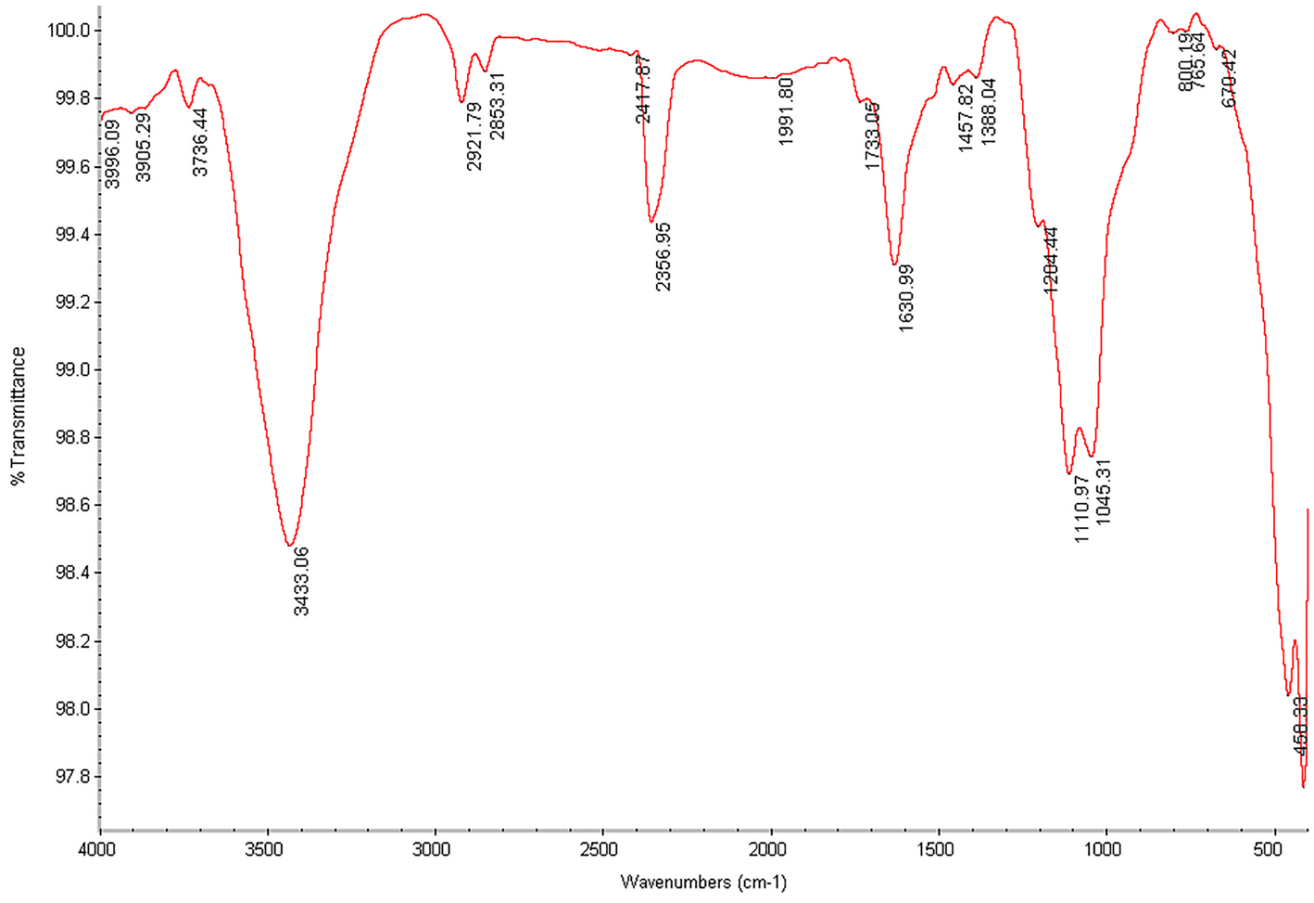
The infrared spectrum of nanotubes functionalized with FLX.

The Fourier Transform Infrared (FTIR) spectrums of carboxylated and drug-functionalized nanotubes are shown in Figs. 1, 2 and 3. The peak at ~ 1525 cm^−1^ corresponds to the C=C double bonds from the nanotube wall. Also, the peaks at 3304 cm^−1^ (Fig. 1) 3437 cm^−1^ (Fig. 2), and 3433 cm^−1^ (Fig. 3) correspond to the carboxylic acid group. The appearance of new peaks with lower wave numbers at ~ 1678 cm^−1^ in Fig. 2 and ~ 1630 cm^−1^ in Fig. 3 were assigned to the amide bond. So The FTIR spectrum of MWCNTs-INH (Fig. 2) and MWCNTs-FLX (Fig. 3) confirmed the formation of amide groups on the MWCNTs surface.

### Scanning electron microscopic results

**Figure 4:**
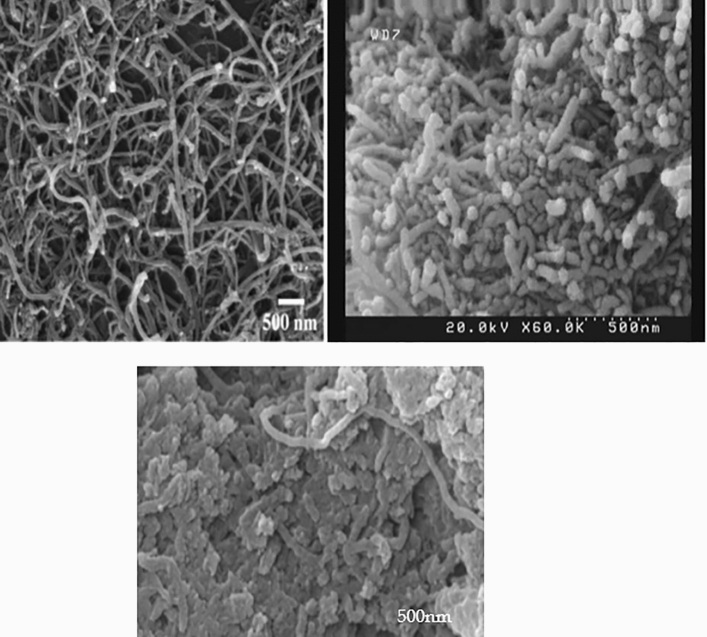
SEM images of the raw MWCNTs (Left), MWCNTs—INH (Right) and MWCNTs—FLX (Below).

Evidence for the functionalization of multi-walled carbon nanotubes can be obtained by SEM electron microscopy images. Figure 4 shows the SEM images of MWCNTs, MWCNTs-INH, and MWCNTs-FLX. As visible in the figures, the drug-functionalized nanotubes have a different morphology and appear rougher compared to the naked nanotubes, which confirms that MWCNTs were functionalized with drugs.

### Results of different types of treatment with INH, FLX, MWCNTs-INH, and MWCNTs-FLX

The effective concentration which could be considered as the MIC value for each treatment is summarized in Table 5. The results obtained by bacterial growth observation for all treatment groups for H37Rv, MDR, and XDR strains are summarized below.

**Table 5:** Summary table of effective concentrations.

Treatment	Strain		
	H37Rv	MDR	XDR
	Concentration that inhibited bacterial growth (µg/mL)		
INH	0.28	1.12	1.12
FLX	1600	1600	1600
INH + FLX	0.56 + 800	0.56 + 800	0.56 + 800
MWCNT-INH	3.125 *(INH 0.26)*	6.25 *(INH 0.52)*	12.5 *(INH 1.04)*
MWCNT-FLX	222 *(FLX 96)*	55.5 *(FLX 24)*	6.93 *(FLX 3.50)*
MWVNT-INH + MWCNT-FLX	0.78 + 55.5 *(INH 0.067* + *FLX 24)*	3.125 + 55.5 *(INH 0.26* + *FLX 24)*	12.5 + 6.93 *(INH 1.04* + *FLX 3.50)*

### H37Rv treatments with the different doses of INH, FLX, INH + FLX, MWCNTs-INH, MWCNTs-FLX, and MWCNTs- INH + MWCNTs- FLX

After incubation with INH for two weeks, a significant decrease in bacterial growth was observed from a dose of 1/1024 (0.14 µg/mL) and no growth was observed from dilutions of 1/512 (0.28 µg/mL) (Figure S1). Also, a significant reduction in growth was observed at a dilution of 1/64 from MWCNTs-INH (1.56 µg/mL containing 0.13 µg/mL INH) and no growth was observed at a dilution of 1/32 (3.125 µg/mL containing 0.26 µg/mL INH) (Figure S2). The efficacy results for FLX indicate that bacterial growth is stopped at dilutions of 40, (1.6 mg/mL) (Figure S3). The treatment results of MWCNTs-FLX showed that no growth was observed from dilution of 2 (222 µg/mL, containing 96 µg/mL FLX) (Figure S4). For Checkerboard treatments on H37Rv, the different concentrations of both drugs from dilution of 2 to 1/4 for MWCNT-FLX and 1/64 to 1/512 of MWCNT-INH were tested. The bacterial growth was significantly reduced when the two nano-drug systems were combined, with dilutions of 1/128 (0.78 µg/mL containing 0.067 µg/mL of INH) of MWCNTs-INH sufficient to prevent bacterial growth when used in combination with the dilution of 1/2 (55.5 µg/mL containing 24 µg/mL of FLX) of MWCNTs- FLX (Figure S5). Checkerboard treatment on H37Rv with FLX and INH was carried out in a previous study and results showed that dilutions of 1/256 of INH (0.56 µg/mL) combined with the dilution of 20 of fluoxetine (0.8 mg/mL) inhibited bacterial growth. The antibacterial effect of MWCNT alone on H37Rv was observed at a concentration of 228 µg/mL (Figure S6).

### MDR & XDR treatments with the different doses of INH, FLX, INH + FLX, MWCNTs-INH, MWCNTs-FLX, and MWCNTs-INH + MWCNTs-FLX

For the MDR strain, INH at dilution of 1/128 (1.12 µg/mL) was required to observe a significant effect on bacterial growth (Figure S7). Similarly for the XDR strain, INH at dilution of 1/128 (1.12 µg/mL) had a significant effect on bacterial growth (Figure S8). Besides, growth of the MDR strain was observed at the dose of 3.2 mg/mL and only has a few growth reductions at 1.6 mg/mL for FLX (Figure S9), while for the XDR strain only a slight growth reduction was observed at this dose of FLX (Figure S10). Results showed that bacterial growth of MDR and XDR strains were been inhibited at the dilution of 1/16 (6.25 µg/mL, containing 0.52 µg/mL of INH) and 1/8 (12.5 µg/mL, containing 1.04 µg/mL of INH) of the MWCNTs-INH respectively (Figures S11, S12).

MWCNTs-FLX has few antibacterial effects on MDR at dilution of 1/2 (55.5 µg/mL, containing 24 µg/mL of FLX) (Figure S13) and also on XDR at the dilution of 1/16 (6.9 µg/mL, containing 3.5 µg/mL of FLX) (Figure S14).

Checkerboard treatment results of MDR and XDR strains with INH and FLX showed that, in the treatment with the dilution of FLX 20 (800 µg/mL) in combination with isoniazid in the dilution of 1/256 (0.56 µg/mL), the bacterial growth was stopped (Figure S15). Besides, in the XDR treatment group at the dose of FLX 40 (1.6 mg/mL) in combination with INH at a dose of 1/128 (1.12 µg/mL) and also at doses of FLX 20 (800 µg/mL) in combination with INH at a dose of 1/256 (0.56 µg/mL), a significant reduction in growth was observed (Figure S16).

After the effect of MWCNTs-INH and MWCNTs-FLX, bacterial growth observation indicated that in the MDR strain, at the dose of 1/2 of MWCNTs-FLX (55.5 µg/mL, containing 24 µg/mL of FLX) in combination with the dose of 1/32 of MWCNTs-INH (3.125 µg/mL, containing 0.26 µg/mL of INH), no growth was observed. In the XDR strain group, the results showed that at doses of 1/16 of MWCNTs-FLX (6.93 µg/mL, containing 3.5 µg/mL of FLX) with a dose of 1/8 of MWCNTs-INH (12.5 µg/mL, containing 1.04 µg/mL of INH), bacterial growth was inhibited (Figure S17).

### Quantitative analyses by Fractional Inhibitory Concentration Index (FICI) for FLX and INH

According to the calculations of the FIC index value for all states of action of isoniazid and fluoxetine in both free and conjugated form with carbon nanotubes, it was shown that in all strains, these two drugs have an additive effect on each other both in free and conjugated forms (Table 6).

**Table 6:** FIC index calculation of treated groups.

Treatment and MIC/µg/mL	INH	FLX	INH + FLX	FIC Index	MWCNT-INH	MWCNT-FLX	MWCNT-INH + MWCNT-FLX	FIC Index
H37Rv	0.28	1600	0.56 + 800	**2.5**	3.125	222	0.78 + 55.5	**0.5**
MDR	1.12	1600	0.56 + 800	**1**	6.25	55.5	3.125 + 55.5	**1.5**
XDR	1.12	1600	0.56 + 800	**1**	12.5	6.93	12.5 + 6.93	**2**

Significant values are in [bold].

### Gene expression analysis and IL6 and TNFα secretion studies

The data analysis of gene expression in various treatment groups was carried out by the One Way ANOVA method and showed that the expression of both *inhA* and *katG* genes in all strains increased significantly in the presence of INH (Figure S18–S20). In the presence of FLX however, both gene expressions were unchanged. For all strains tested, every treatment group involving INH saw a sharp increase in *inhA* expression, irrespective of the presence of FLX or the formulation in MWCNT. In contrast, the expression of *katG* was significantly reduced by combination therapy with free FLX, and the conjugated form of INH led to weaker levels of expression (Figure S18–S20). Results of the cytokine secretion studies showed that the levels of IL6 and TNFα secretion were significantly increased in all the treated groups, indicating that the drug treatment was able to induce pro-inflammatory pathways in TB-infected macrophages (Figure S21–S23), presumably due to efficient killing of the bacteria. The secretion of IL6 and TNF-α cytokines from TB-infected macrophages were similar between free and MWCNT-conjugated drugs, indicating that the CNT vectorization strategy could maintain an efficient targeting of the intracellular bacteria.

## Discussion

In this study, the main focus was on comparing the efficacy of nano-type drugs with that of the pure, free drugs and evaluating the combined effects with host targeting agents like FLX, belonging to the group of serotonin reuptake inhibitors (SSRIs). INH is still one of the most active anti-tuberculosis agents, used for both the treatment and prevention of TB. Although INH is one of the safest and most cost-effective drugs for the treatment of TB, it causes many side effects in patients with high and long-term doses^7^.

Selective serotonin reuptake inhibitors are antidepressants and have shown significant activity against bacterial resistance, but the antibacterial effects, as well as the antibiotic modulatory properties of FLX, are not yet clear. The results of previous studies showed that SSRIs drugs have significant antimicrobial activity against gram-positive bacteria, but are ineffective against Enterobacteria other than Citrobacter and *Pseudomonas aeroginosa*^33^. In this study, the mentioned effects of FLX were confirmed against different strains of Mtb^34^, including the MDR and XDR strains. Additional studies are needed to describe the antimicrobial properties of this drug as well as the clinical implications of its use in the treatment of infections by resistant microorganisms. On the other hand, the molecular mechanisms of INH resistance include several genes that are involved in several biosynthetic networks and pathways. Mutations in the *katG* and *inhA* genes are major factors in INH resistance, followed by *ahpC, kasA, ndh, iniABC*, and *fadE*^35,36^. Therefore, in this research, the expression of these two main genes (*inhA, katG*) was studied before and after drug treatments by Real-Time PCR. The results of this study showed that the expression of *katG* was significantly reduced by combination therapy with both free and conjugated drugs.

As a result of a study in 2018 on a large number of CNS drugs, a synthetic analog of FLX was introduced as a selective and specific anti-tuberculosis agent and the antibacterial effects of this drug on the standard strain of tuberculosis (H37RV) were tested^37^. The results of some research showed that the use of a series of small molecules called Host-targeted inhibitors could target TB-infected macrophages and stop the growth of Mtb. The use of drugs such as FLX, a selective serotonin reuptake inhibitor, can increase the secretion of pro-inflammatory cytokines, induce autophagy in TB-infected macrophages and stop the growth of Mtb^38,39^. However, extensive studies on the toxicity of these nanostructures need to be conducted. Targeting the host is one strategy considered in this study. Also, in this research usage of FLX which is belonging to the group of serotonin reuptake inhibitors (SSRIs) has shown significant antimicrobial activity.

On the other hand, nanotubes can be activated with groups that give them new properties so that they can be easily used in the environment. With the help of nanotubes and their attachment to drug compounds such as INH and FLX by chemical reactions, a new nano product was prepared. We showed here that these new compounds have equivalent biological activity and could be classified in the family of nano-drugs^3,24,40,41^. Since anti-tuberculous drugs usually cannot target macrophages and have a low concentration in the lesion, so they cannot successfully kill *mycobacterium tuberculosis*. In addition, these drugs have no efficient penetration in the cell wall of the bacteria because of their unique cell envelope structure and composition, containing a peptidoglycan layer and its drug resistance mechanisms. In the study, we constructed a CNT-based drug delivery system, which directs the drug to the target area as well as effectively destroys the bacterial cell wall. Even in recent studies was found that the outstanding application of nano-drug delivery systems in this way is to target infected macrophages or macrophage-targeted delivery systems for enhanced intracellular *Mycobacterium tuberculosis* killing efficiency^42^.

**Figure 5:**
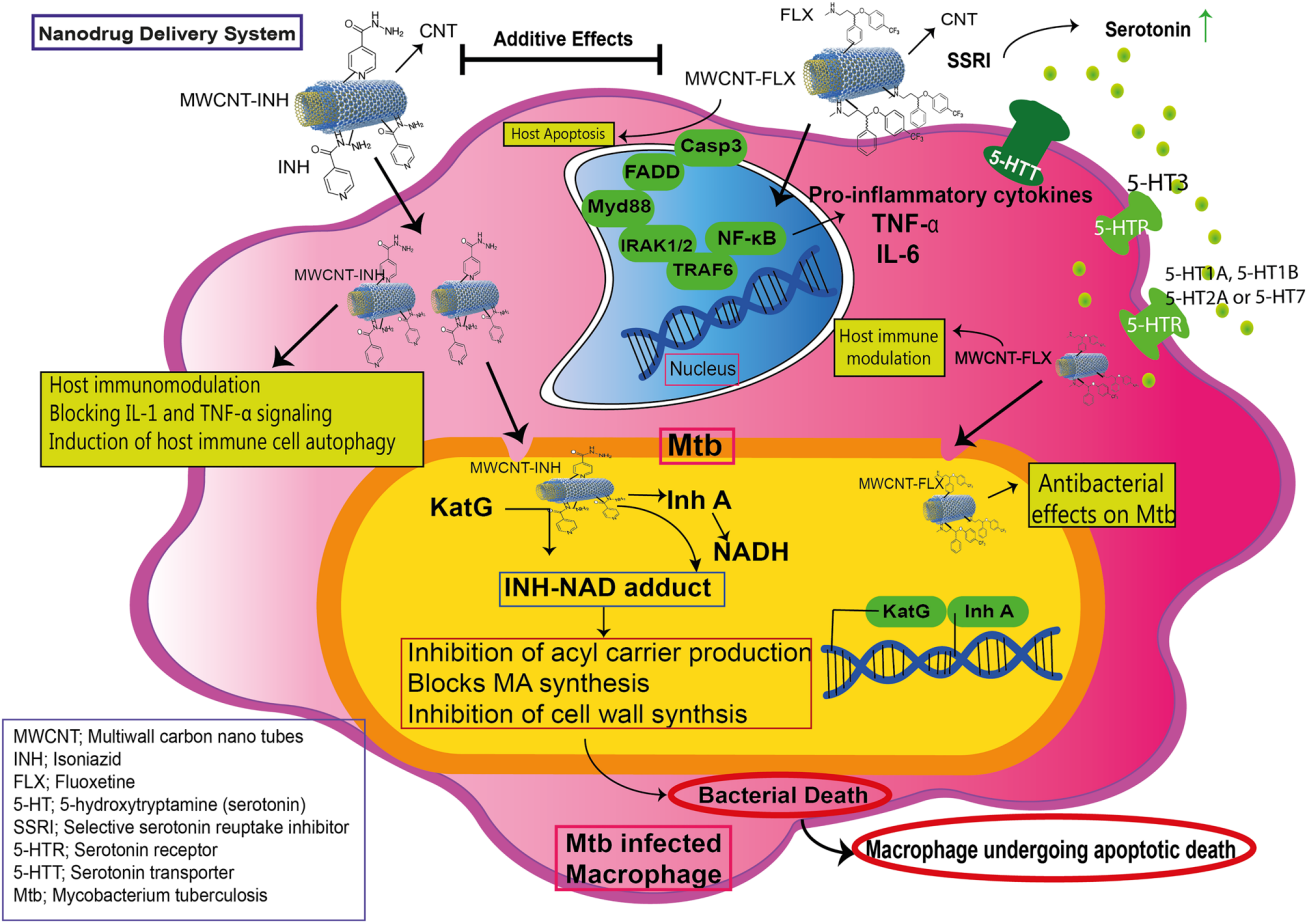
The chemical structures, mechanism of drug release and signaling pathway of the MWCNTs conjugated with drugs.

It is suggested that the uptake of functionalized MWCNTs performs by two ways, first direct penetration via the plasma membrane, and second, endocytosis processes^25^. Therefore, it seems that CNTs have several function in drug delivery including improve cellular uptake via disruption of bacterial cell wall, optimization of release rate, and less toxicity for host cells^43^. Figure 5 depicted the chemical structures of the MWCNTs conjugated with drugs and also the mechanism of drug release from MWCNTs.

## Conclusion

In this study, using MWCNTs, a new form of the antibiotic INH and the drug FLX were made to improve antibacterial activity by taking advantage of the additive effect that these two drugs have on each other. However, the results of this investigation, confirmed previous reports that the use of serotonin receptor agonists or antagonists can activate the autophagy pathway to kill TB bacteria, more detailed and complete studies on the relevant signaling pathways are necessary.

Because the process of approving a drug is time-consuming and costly, these methods can be obtained sooner through combination therapies because these drugs are known for their pharmacokinetic profile, and safety, are cheaper, more accessible, and can enter Phase 2 of the clinical trial faster. Also, with the increase in the number of people affected with MDR-TB, XDR-TB, and so-called drug-resistant tuberculosis (TDR-TB), new treatment strategies are needed that go beyond the conventional antibiotics.

## Supplementary Information

Supplementary Information.

## Data Availability

All data generated or analysed during this study are included in this published article (and its supplementary information file).
