# Supplementary material for: An effective nano drug delivery and combination therapy for the treatment of Tuberculosis
Source: Sci Rep. 2022 Jun 10;12:9591. doi: 10.1038/s41598-022-13682-4 (PMC9185718; doi:10.1038/s41598-022-13682-4)
Supplement: Supplementary file 1 — Supplementary Information. [file 41598_2022_13682_MOESM1_ESM.docx]

*
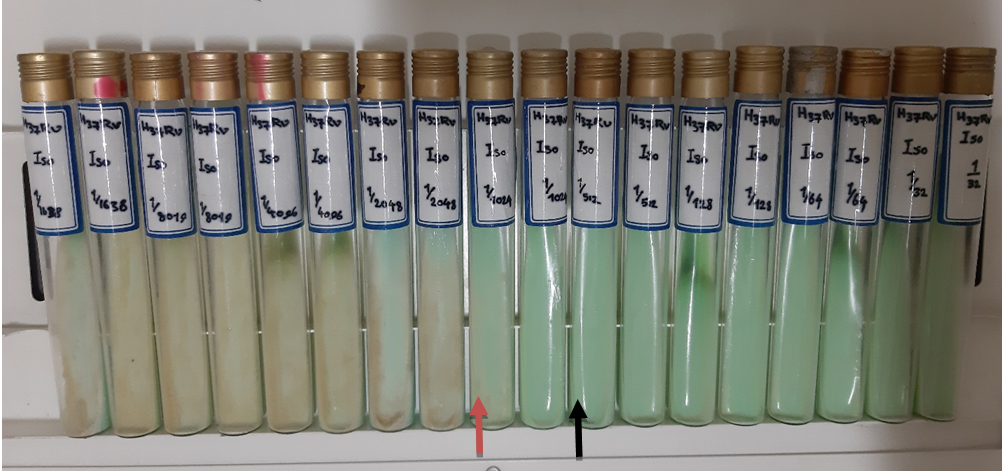
*

**Figure S1**. Results of the effect of INH on the standard strain of *Mycobacterium tuberculosis* H37Rv. According to the figure, at a dose of 1/1024, a decrease in growth was observed (orange arrow), at a dose of 1/512, complete absence of growth was observed (black arrow).


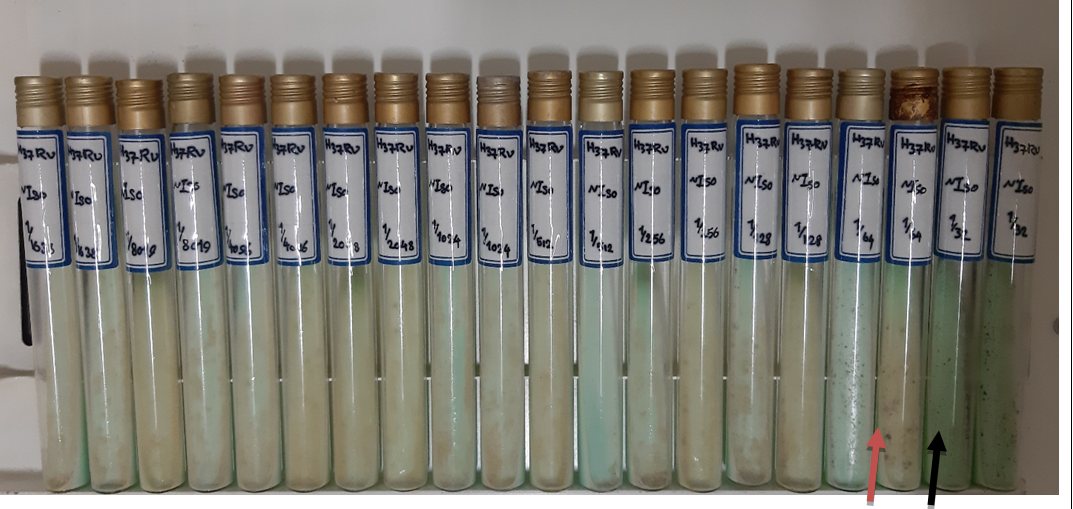


**Figure S2**. Results of the effect of MWCNTs-INH on H37Rv. A reduction of growth was observed at a dilution of 1/64; inhibition of growth occurred at a dilution of 1/32.


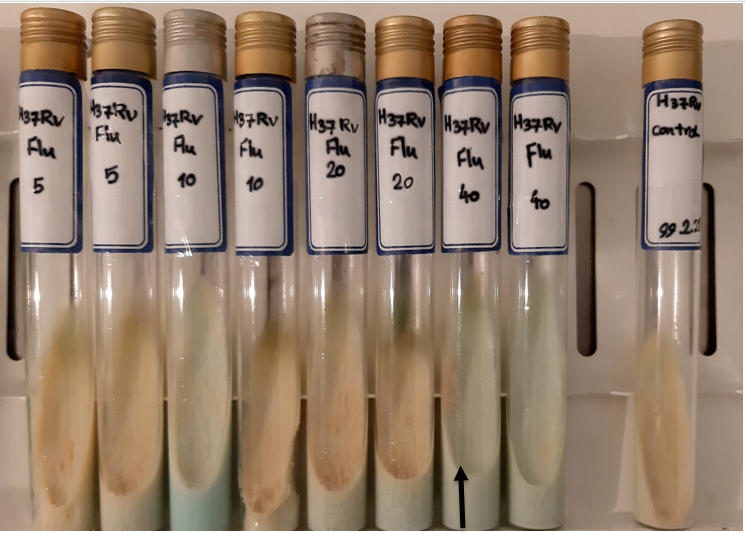


**Figure S3**. As shown above, with effect of fluoxetine on the H37Rv strain, at dose of 40 (1.6 mg/mL), a significant reduction in growth was observed


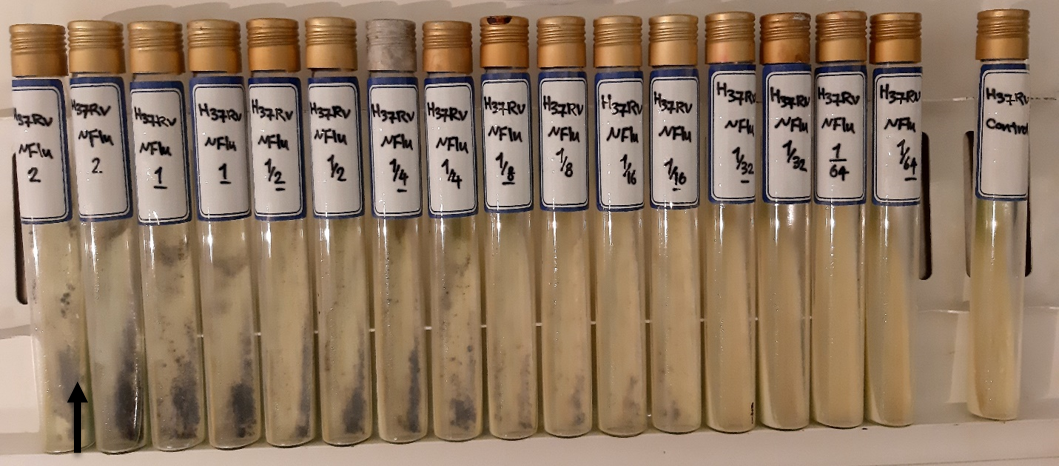


**Figure S4**. Results of the effect of MWCNTs-fluoxetine on the standard strain of *Mycobacterium tuberculosis* H37Rv. No growth was observed from dilution of 2 (222µg/mL of conjugated CNT with drug containing 96 µg/mL FLX).


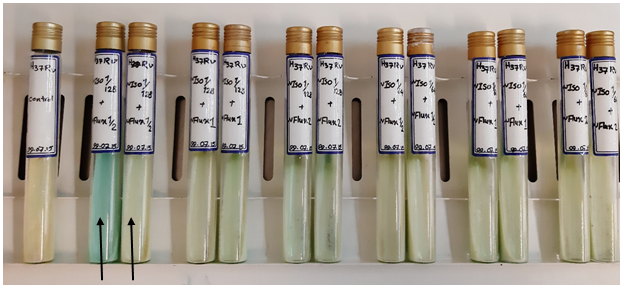


**Figure S5**. Results of the MWCNTs-fluoxetine and MWCNTs-INH effects on H37Rv via Checkerboard treatment studies.


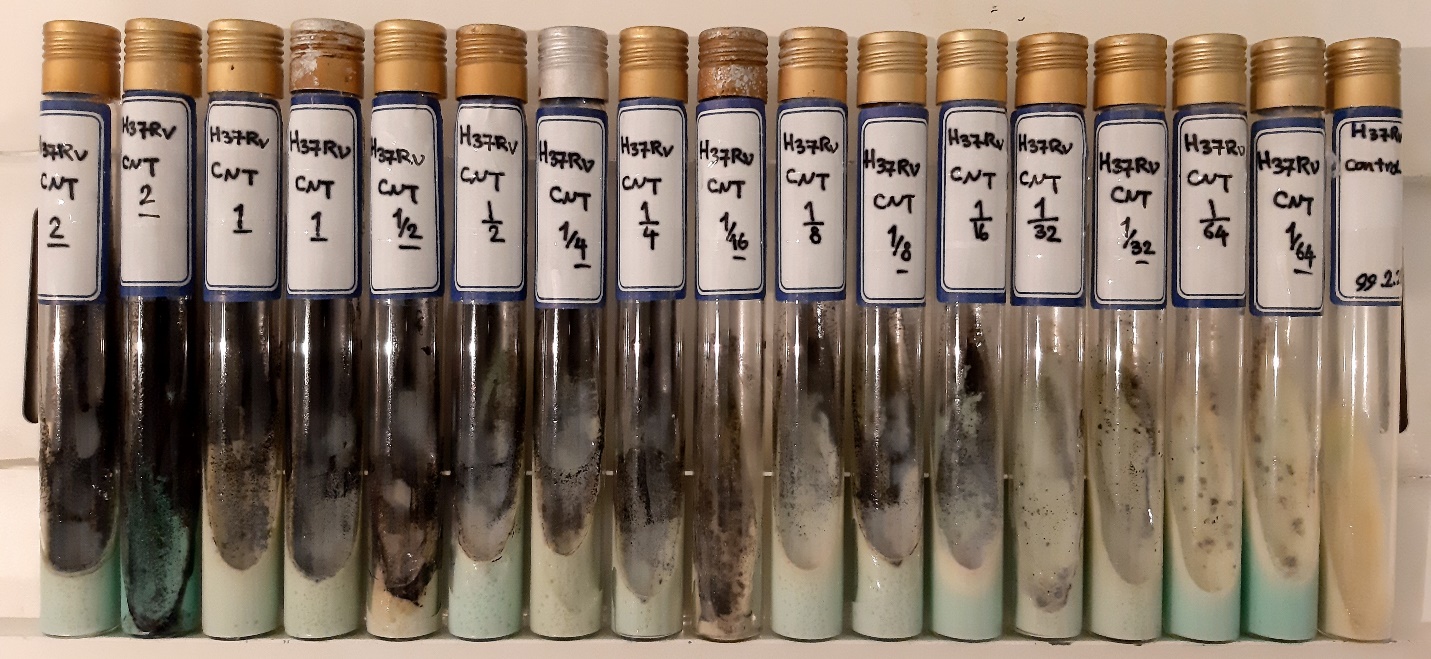


**Figure S6**. The effect of MWCNT alone on *H37Rv*. According to the figure, at the concentration of 114µg / mL, growth reduction was observed and at the concentration of 228 µg / mL, no growth was observed


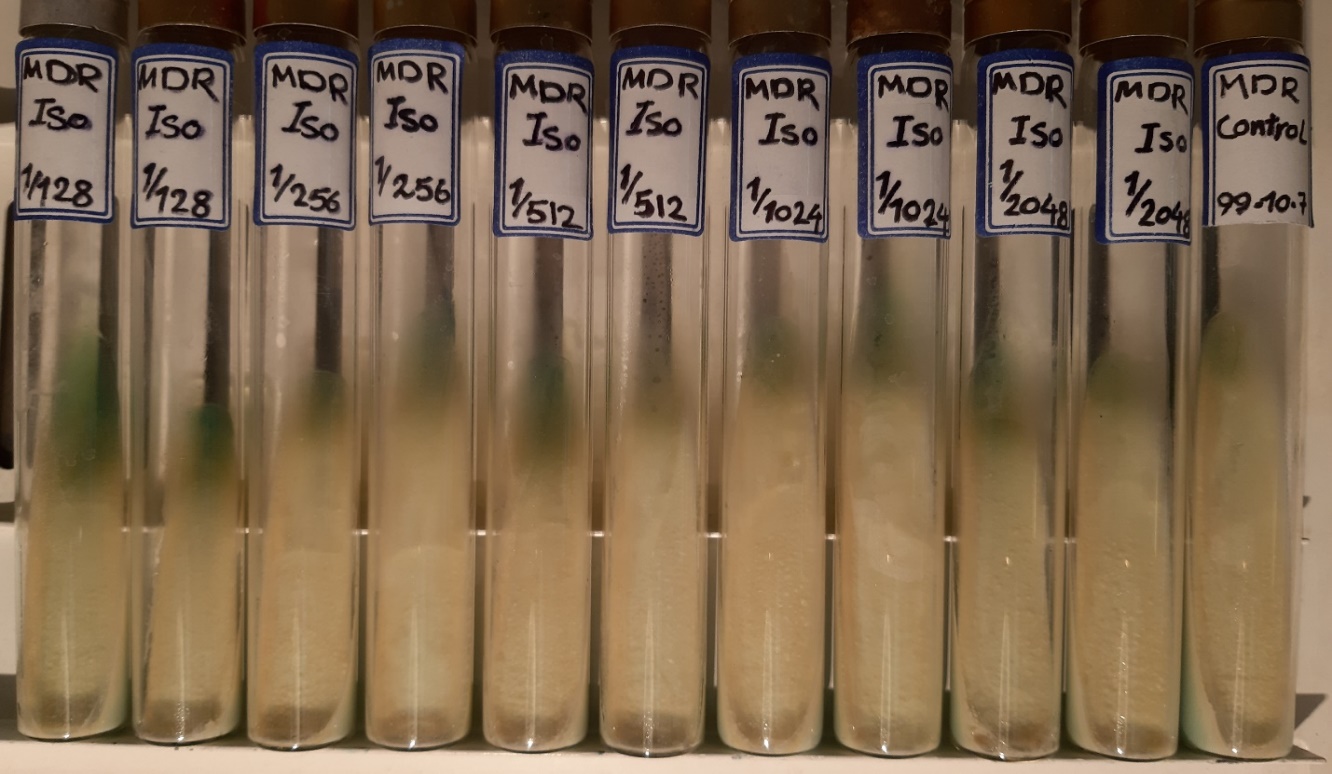


**Figure S7**. Results of the *INH* effects on MDR strain. Bacterial growth was decreased at the dilution of 1/128

**
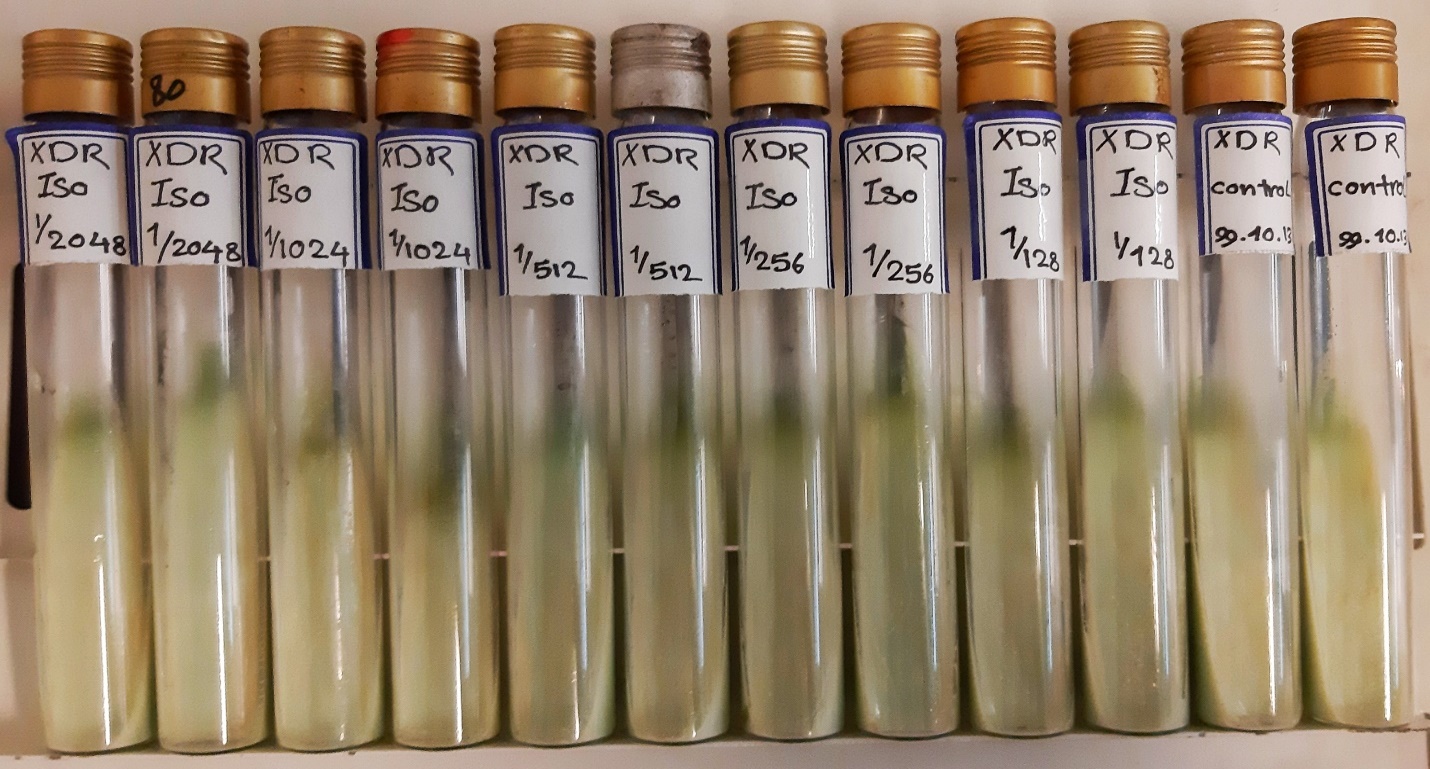
**

**Figure S8**. Results of the *INH* effects on XDR strain. Bacterial growth was decreased at the dilution of 1/128

**
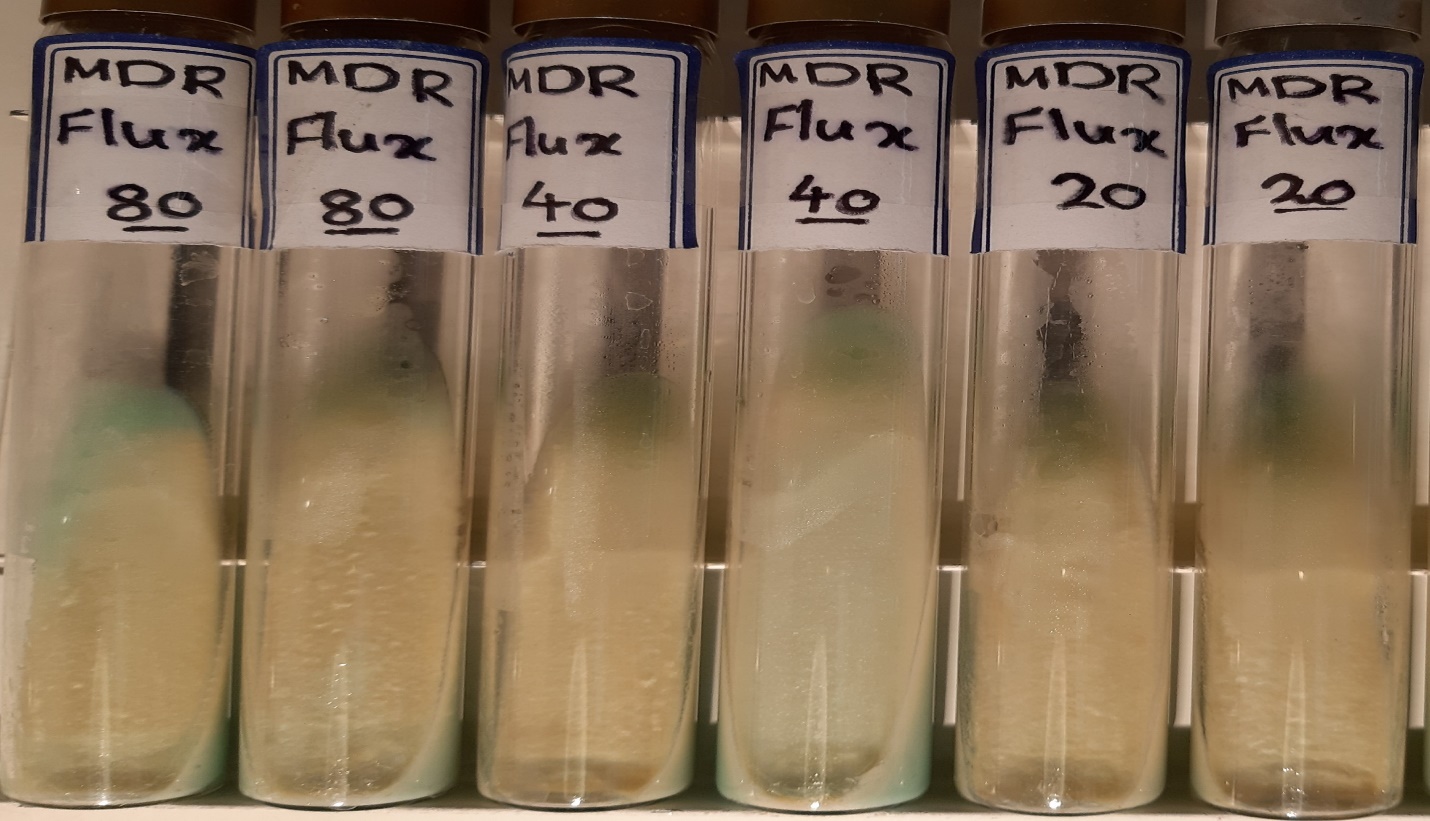
**

**Figure S9**. Results of the Fluoxetine effect on MDR strain.

**
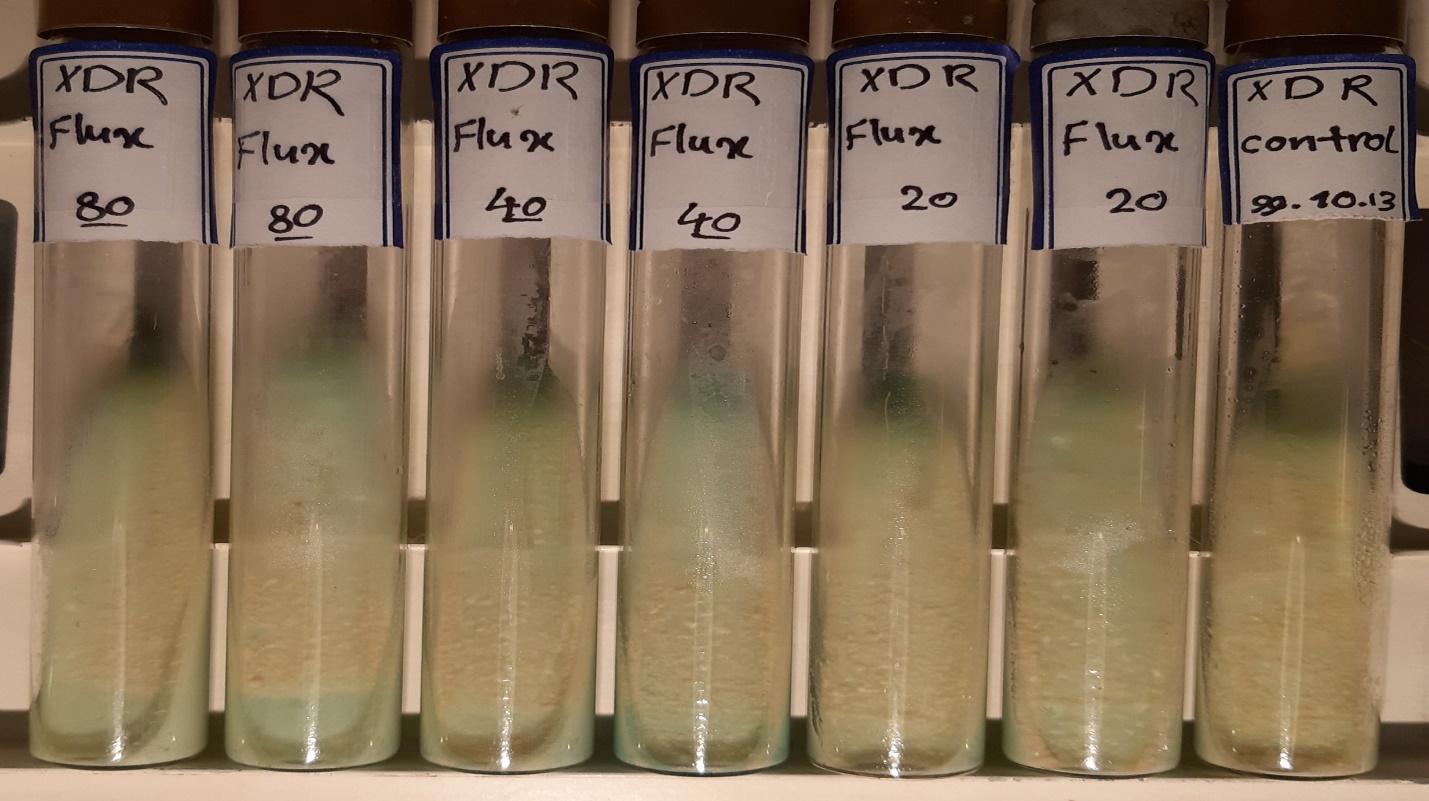
**

**Figure S10**. Results of the Fluoxetine effect on XDR strain.

**
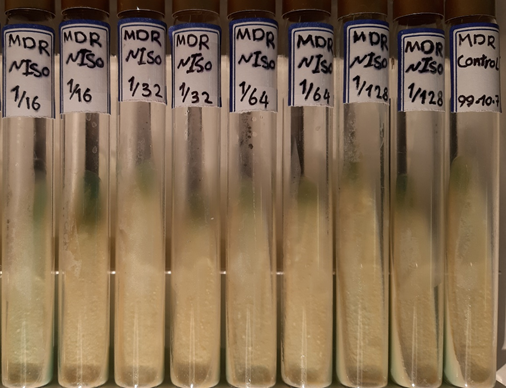
**

**Figure S11**. Results of the MWCNTs-*INH* effects on MDR strain. Bacterial growth was been inhibited at the dilution of 1/16


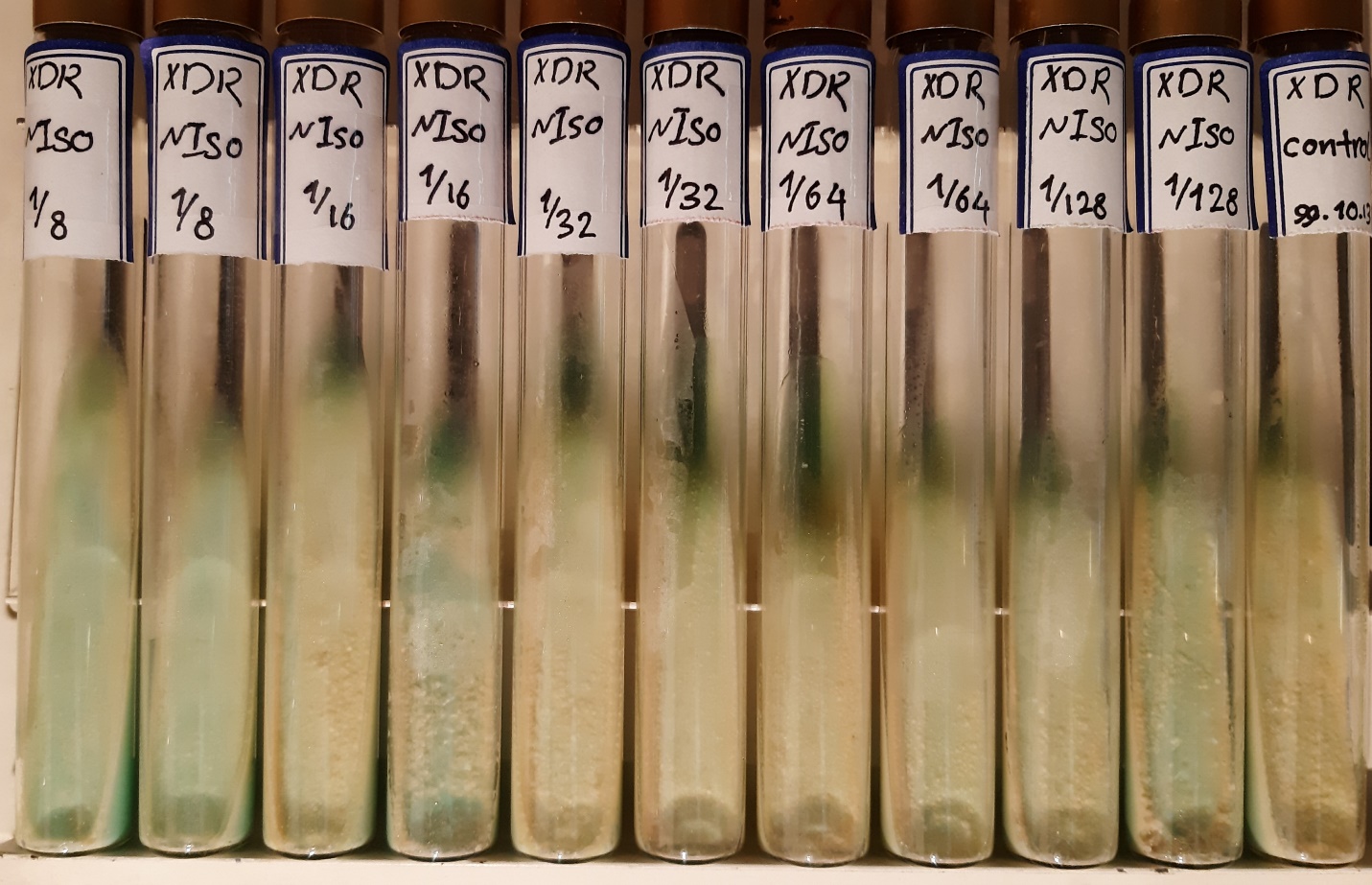


**Figure S12**. Results of the MWCNTs-*INH* effects on XDR strain. Bacterial growth was decreased at dilution of 1/128 and stopped at the dilution of 1/8


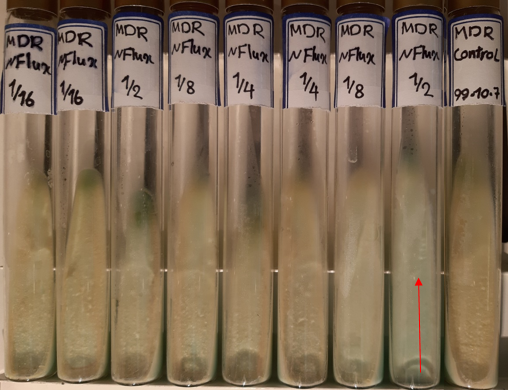


**Figure S13**. Results of the MWCNTs-Fluoxetine effect on MDR strain.


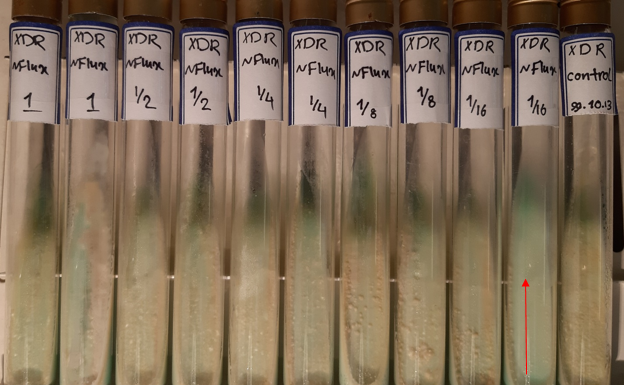


**Figure S14**. Results of the MWCNTs-Fluoxetine effect on XDR strain.


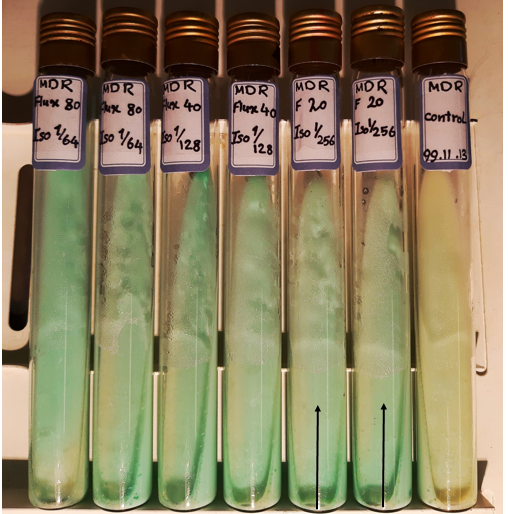


**Figure S15**. Checkerboard treatment results of MDR strain with INH and fluoxetine


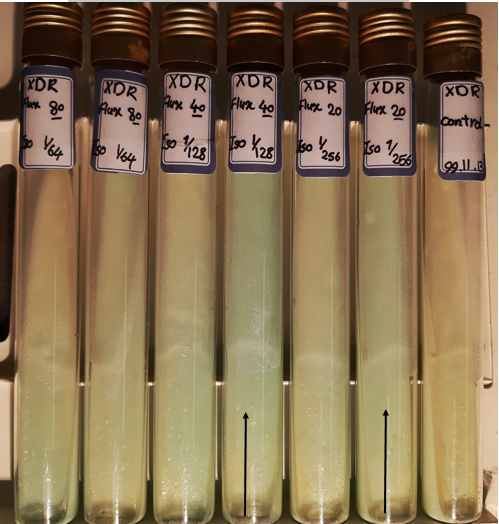


**Figure S16**. Checkerboard treatment results of XDR strain with INH and fluoxetine


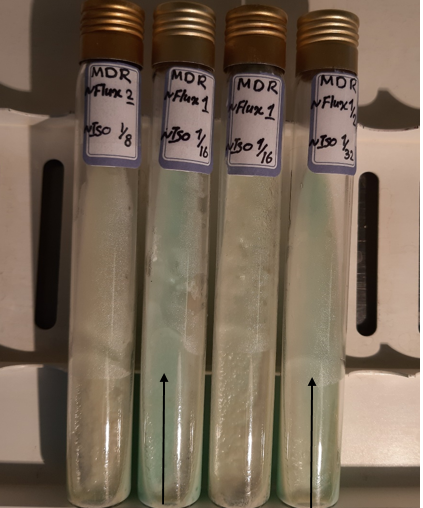

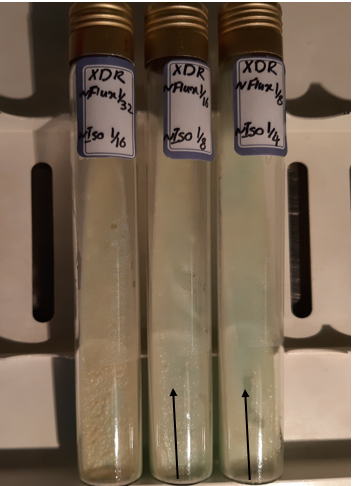


**Figure S17**. Checkerboard results of MDR (left) and XDR (Right) strains treatments with *MWCNTs-INH and MWCNTs-fluoxetine*

***
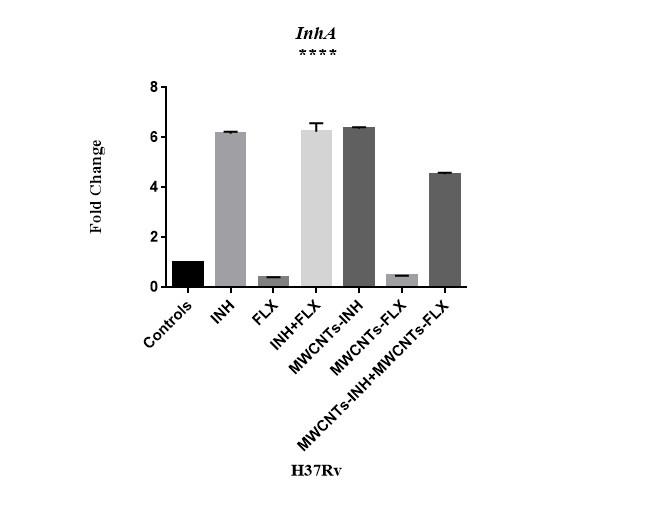

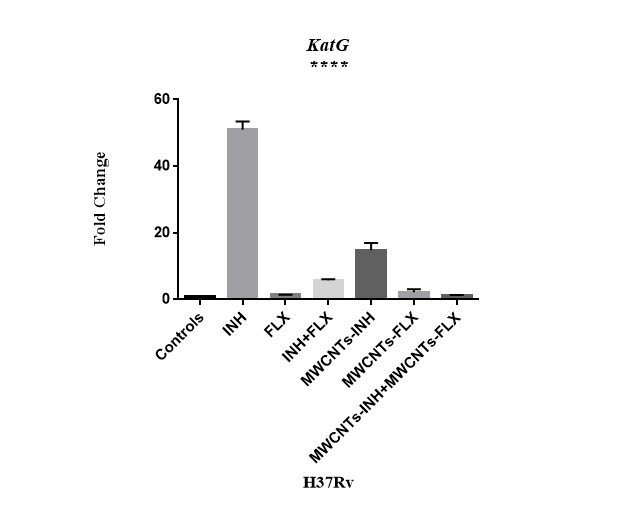
***

**Figure S18**. Comparison charts of *inhA* and *katG* genes expression in drug-treated groups in H37Rv strain

***
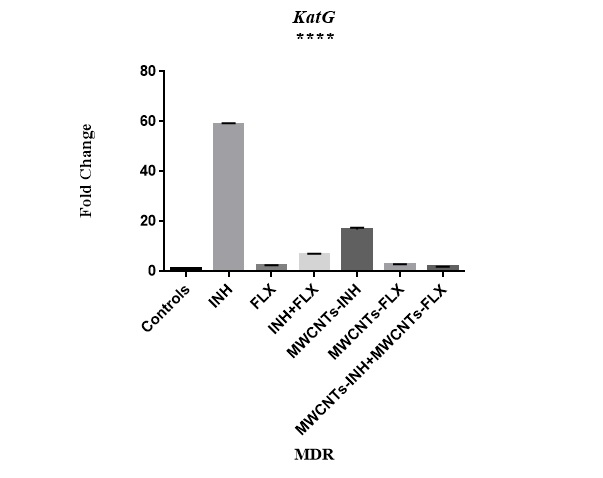

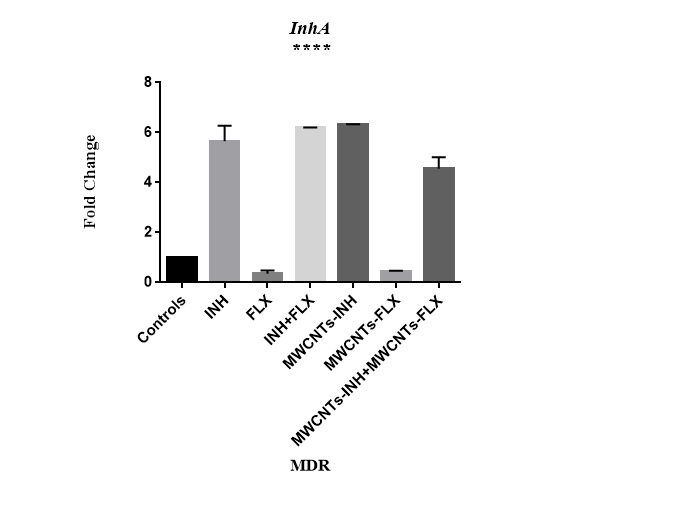
***

**Figure S19**. Comparison charts of InhA and KatG genes expression in drug-treated groups in MDR strain

***
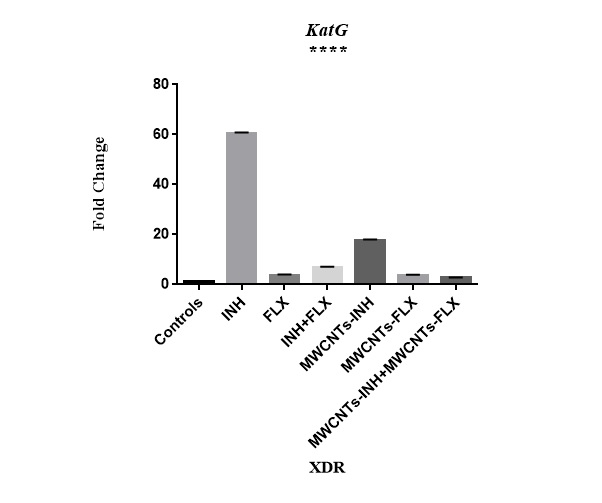

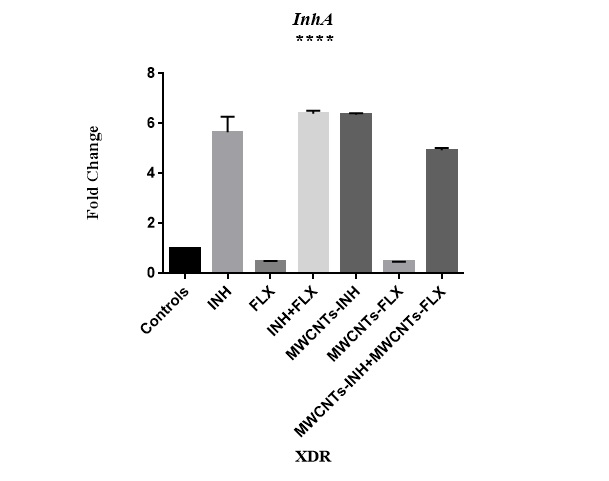
***

**Figure S20**. Comparison charts of InhA and KatG genes expression in drug-treated groups in XDR strain


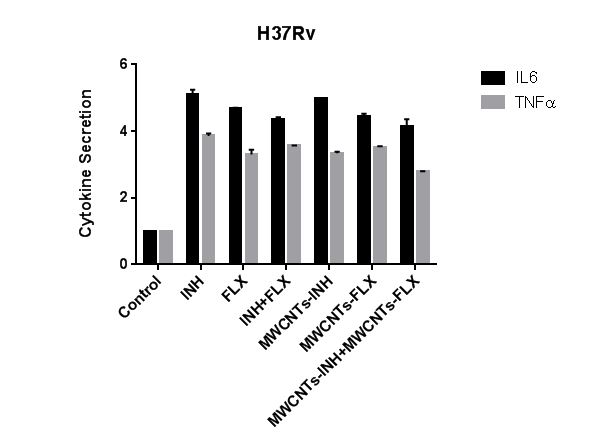


**Figure S21**: The secretion value of IL6 and TNF-α cytokines related to TB-infected macrophages with H37Rv strain after treatment with different groups.

**
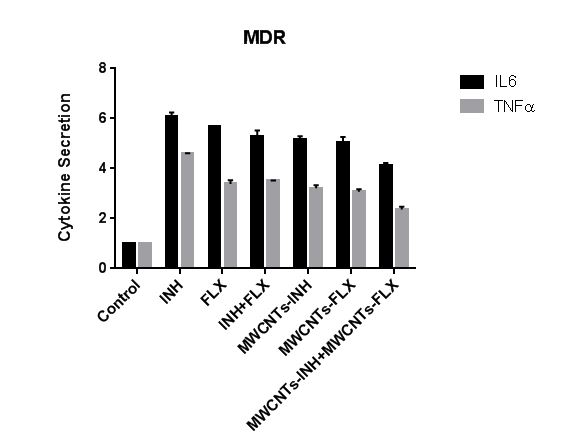
**

**Figure S22**: The secretion value of IL6 and TNF-α cytokines related to TB-infected macrophages with MDR strain after treatment with different groups.

**
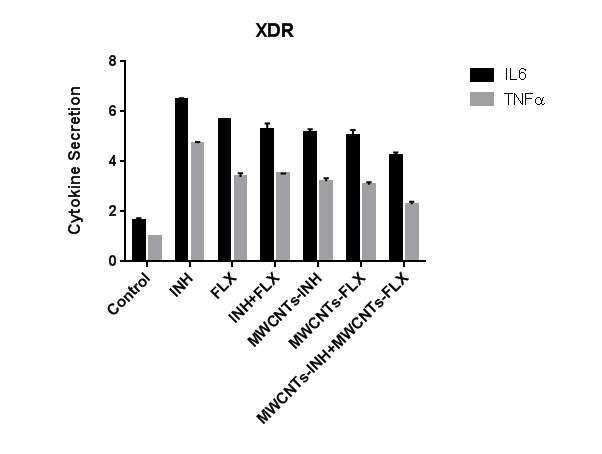
**

**Figure S23**: The secretion value of IL6 and TNF-α cytokines related to TB-infected macrophages with XDR strain after treatment with different groups.
